# New Classification of Midpalatine Suture Maturation Using Cone Beam Computed Tomographic Study

**DOI:** 10.3390/diagnostics15222925

**Published:** 2025-11-19

**Authors:** Cristalle Soman, Reem Khaled Alshammari, Nawal Mohammad AlMutairi, Lolwah Mohammad Alenezi, Rayan Alaadwany, Mohammad Abdul Baseer, Fahdah Aldahash, Malak AlOsaimi, Sara Tarek Ahmed, Nancy Ajwa, Yasmine Tarek Ahmed

**Affiliations:** 1 Department of OMFS & Diagnostic Sciences, College of Medicine and Dentistry, Riyadh Elm University, Riyadh 11681, Saudi Arabia; 2 College of Medicine and Dentistry, Riyadh Elm University, Riyadh 11681, Saudi Arabia; 3 Preventive Dentistry Department, College of Medicine and Dentistry, Riyadh Elm University, Riyadh 11681, Saudi Arabianancy.ajwa@riyadh.edu.sa (N.A.); 4 Department of Prosthodontics, College of Dentistry, Riyadh Elm University, Riyadh 11681, Saudi Arabia; 5 Restorative Dentistry Department, College of Medicine and Dentistry, Riyadh Elm University, Riyadh 11681, Saudi Arabia

**Keywords:** palatal expansion, midpalatine suture, cone beam computed tomography, cranial sutures, palatine bone, rapid maxillary expansion

## Abstract

**Background/Objectives:** Assessment of the midpalatine suture is vital for making clinical decisions regarding the correction of transverse growth discrepancies of the maxilla. Several studies have used Cone Beam Computed Tomography (CBCT) to evaluate skeletal maturity by midpalatine suture staging (MPS) in various populations. A few patterns of staging did not fit the standard classification. Hence, the rationale of this research was to explore potential new subcategories of maturation staging using CBCT. The study aimed to develop a new comprehensive classification subcategorization system for midpalatine suture maturation staging based on CBCT scans. **Methods**: The study involved the retrospective analysis of 168 CBCT scans. The standard reference for MPS staging was taken from a previous published classification in 2013 using CBCT. Each classification stage of the standard classification was subcategorized into Pattern A and Pattern B. **Results:** Both classifications (standard reference and new) can rely on age to predict the possibility of maturation of the MPS compared to non-maturation. Age is a predictable variable of suture opening in both classifications. **Conclusions:** The new classification demonstrated increased sensitivity in detecting midpalatine suture maturity and also increased the likelihood of utilizing non-surgical maxillary expansion compared to the previous classification. Evaluating suture staging in individual cases using CBCT is recommended for personalized diagnosis and optimal treatment planning for maxillary expansion. This advancement allows clinicians to use the new classification as a reliable tool to confidently predict non-surgical expansion success for more mature patients, thereby broadening the scope of orthodontic treatment without compromising patient outcomes.

## 1. Introduction

During growth, a series of joints grows at the boundaries of the adjacent bones of the cranium, mainly belonging to a type of syndesmosis defined as ‘suture’. Sutures permit stress dispersal and bone remodeling through growth, which occurs over the period of bone formation during sutural distraction [1]. The midpalatine suture is known as the junction of two bones that meet at the same end and undergo changes in their morphology as they grow. Its shape is described as a Y shape in the frontal part [2].

The assessment of the development of the mid-palatine suture is extremely vital before a clinical decision on correction of a transverse growth discrepancy of the maxilla, termed as slow maxillary expansion (SME), rapid maxillary expansion (RME) with a conventional palate expander in cases with opened suture (children and adolescents) or surgically using MARPE (mini screw-assisted rapid palatal expander) and SARPE (surgical assisted rapid palatal expander) in closed suture cases such as adult patients [3]. Cone Beam Computed Tomography (CBCT), being an advanced imaging modality in dentistry, can be used to study the growth patterns of the midpalatine region.

Mid-Palatine Suture (MPS) development was studied using CBCT in different populations to evaluate skeletal maturity [2,4,5,6]. These studies aimed to classify the different stages of midpalatine suture fusion, which is crucial in determining the optimal timing for treatments involving maxillary bone expansion, particularly in adolescents and young adults [5]. Notably, previous studies by Haghanifar et al. (2017) and Soman et al. (2025) found new observed patterns of MPS staging, other than those mentioned by the previous classification [2,4,7].

Interestingly, age is usually correlated with suture maturation. It is expected that the suture will be closed entirely at a certain age. However, variations in suture closure can exist within the same bone, mainly when the bone is formed from more than one component, like the palatine bone that has the maxillary part (premaxilla) and the palatine part. There are no reported studies that have explored the possibilities of new subcategories beyond the standard classification by the previous classification [4]. The current lack of detailed sub-staging may contribute to inconsistent diagnostic interpretations and sub-optimal treatment planning in cases that require maxillary expansion fine ambiguous radiographic presentation. Hence, the rationale of this study was to explore the possibilities of identifying varying patterns in the maturation staging of MPS, aiming to provide a more detailed and clinically relevant assessment tool.

The aim of the study was thus to develop a comprehensive subcategorization system for midpalatine suture maturation staging based on Cone Beam Computed Tomography. The null hypothesis of the study was that there is no significant difference between midpalatine suture maturation staging using CBCT and the previous reference classification by and staging using the proposed new classification.

## 2. Materials and Methods

*Study design*: Retrospective cross-sectional study on CBCT scans.

*Study Setting*: The study was conducted in a private dental university affiliated with the investigators. Retrospective CBCT scans were collected (specification-Sirona Galileos, Germany, Bensheim city at 85 kV, 57 mA, 14s) between January 2018 and January 2024. The study was conducted in accordance with the Declaration of Helsinki 2013, and approved by the Institutional Review Board (or Ethics Committee) of Riyadh Elm University (IRB no. FUGRP/2024/368/1162/1045 on 8 October 2024) and a waiver of informed consent for the analysis of retrospective data was approved.

*Participants/Samples*: CBCT scans were screened following the eligibility criteria below.

Inclusion Criteria:

- Both genders.
- CBCT scans with clear images of the region of interest.

Exclusion Criteria:

- Impacted teeth in the region of interest.
- History of or ongoing orthodontic treatment or use of orthodontic appliances.
- History of maxillofacial trauma or surgery in the maxilla.
- Presence of systemic diseases affecting the bone or bone diseases.
- Presence of pathologies, developmental abnormalities, or syndromic conditions affecting the maxilla.
- CBCT scans with noise or blurred images.

*Sampling*: Samples were segregated during data analysis using a stratified random sampling technique, where age served as the basis for stratification. The stratified sampling method was used to ensure that all biological maturation stages, from early adolescence to young adulthood, were included in the study sample. This approach enabled the new classification to be tested across various age groups where the MPS varies, and the growth stage can be harnessed for maxillary expansion, allowing for increased precision in this process. Randomization was subsequently carried out within each stratified group to recruit samples into the study, ensuring unbiased representation of the study sample and facilitating statistical validity. The stratified samples included four groups: 10–13 years (early stage of adolescence), 14–17 years (mid-adolescent stage), 18–21 years (late adolescent stage), and 22–25 years (young adulthood). After segregation, random sampling was conducted from these groups.

*Sample size*: The sample size calculation was derived from a previous study by Jimenez-Valdivia et al. (2019) [5]. The sample size parameters of Jimenez-Valdivia et al.’s 2019 study provided the statistical rationale for validating the new classification in the quest to detect an open MPS (one proportion) in each category or stratum of the sample. This calculation increased the likelihood of managing maxillary expansion non-surgically. Thus, by using this sample size calculation, it was ensured that this study had adequate samples to measure this one proportion reliably with a precision of 5%, confidence interval 95%, 80% power, and an anticipated proportion of 10% (which guaranteed to detect even a few samples in a group with clinical significance); the minimum required sample size was 163 CBCTs. The current study gathered 168 CBCT scans, applied the new classification, and compared it to the previous standard classification regime [2].

*Variables*:

- *Independent Variables*: Age, Gender, stages of maturation of the MPS.
- *Dependent Variables*: Open or Fused MPS (open sutures are non-surgically expandable and fused being surgically expandable), prediction of possibility of open MPS according to the new classification.
- *Covariates*: Age and Gender.

*Data sources/measurement*: The analysis of the CBCT images was done using Galileos viewer (Sidexis XG), version 1.6. The sample CBCT images were viewed using multiplanar reformatted windows, allowing for detailed analysis of the axial, coronal, and sagittal planes. The skeletal stages of maturation of the midpalatal suture were visualized and classified using axial sections following the reference staging below and further scrutinized for a newly proposed subcategorization of maturation of the MPS. All of the patient identifiers were removed to anonymize the data after the data collection was completed.

*Reference Staging for the Maturation of Midpalatine Suture* [2]

Stage A: Nearly straight high-density sutural line with no or minimal interdigitation in the maxilla and palate.

Stage B: One or two irregular, high-density, scalloped sutural lines in the maxilla and palate. When two lines are present, they may be parallel with a few small low-density radiolucent spaces in between the lines.

Stage C: Straight or irregular, two well-defined high-density lines in the maxilla and palate, scalloped with small low-density intra-sutural spaces.

Stage D: Fusion of the palatal suture with bone, while maxillary suture is still two high-density lines with radiolucent spaces in between.

Stage E: Fusion of mid-palatine suture in the maxilla and palate where no visible suture is identifiable.

**Figure 1:**
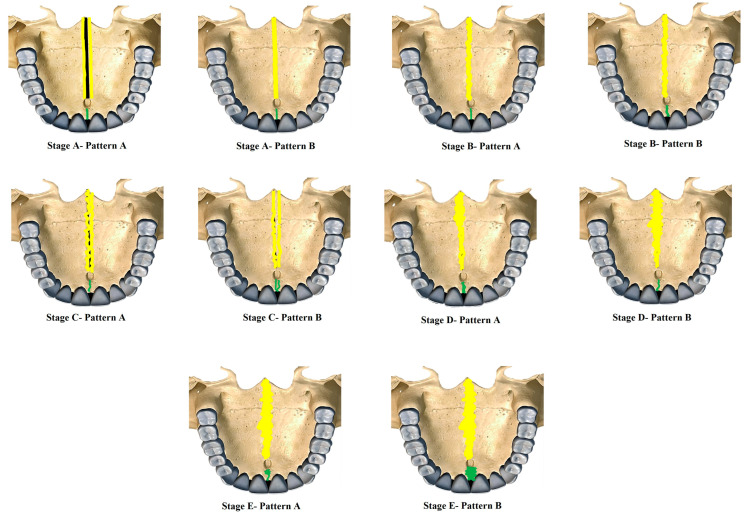
New classification of Midpalatine Suture Staging with five stages and two patterns per stage represented in skull based pictorial representation. Yellow lines represent the palatine part and green line represents the maxillary/premaxillary part of Midpalatine suture.

**Figure 2:**
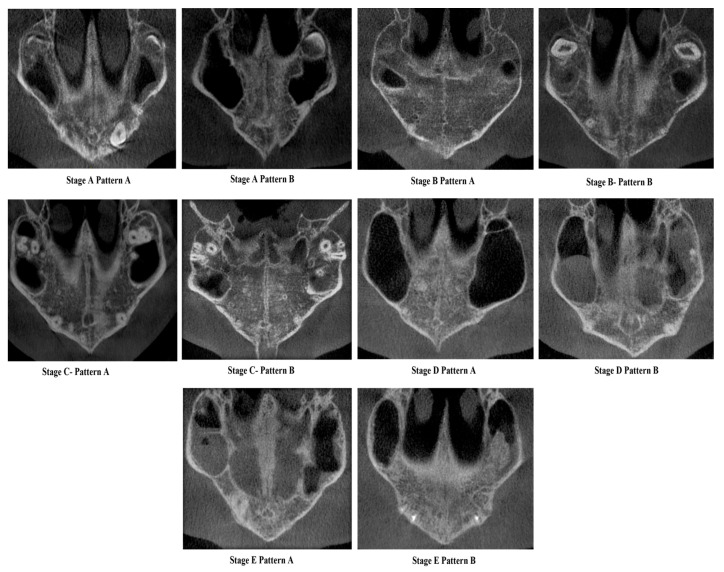
Cone Beam Computed Tomographic images of the New classification of Midpalatine Suture Staging with five stages and two patterns per stage.

*Proposed new classification* [Figure 1 and Figure 2]

Stage A—Pattern A—Single, wide, continuous radiolucent linear space bounded by well-defined radiopaque lines extending from posterior palate anteriorly.

Stage A—Pattern B—Same as the previous standard classification Stage A—Single high-density relatively straight radiopaque (RO) line [2].

Stage B—Pattern A—Single straight high-density sutural line in the maxillary region, comparable to Stage A. The suture is single scalloped toward the palatal bone region.

Stage B—Pattern B—Equivalent to the previous standard classification Stage B—Single scalloped line of high-density radiopacity.

Stage C—Pattern A—Single scalloped line in the region of the premaxilla and toward the palate with two parallel RO lines with small radiolucent (RL) areas.

Stage C—Pattern B—Similar to the previous standard classification Stage C—Continuous or irregular-2 RO, non-scalloped or scalloped, parallel lines separated by low-density (RL) areas in the palatal and premaxillary region.

Stage D—Pattern A—Palatine suture (not completely fused)—50% or less than 50% fused in the posterior maxilla and continues anteriorly as two parallel radiopaque straight or irregular lines with radiolucent spaces, maxillary suture seen as two continuous or irregular RO lines or a single radiopaque line.

Stage D—Pattern B—Palatine suture—more than 50–100% fusion of posterior palatine region and continues anteriorly as straight or irregular lines with radiolucent spaces in between, maxillary suture seen as two continuous or irregular RO lines or a single radiopaque line.

Stage E—Pattern A—Palatine suture completely fused, but maxillary suture 50% or less than 50% fused and seen as two RO lines toward maxilla.

Stage E—Pattern B—Palatine suture completely fused and maxillary suture more than 50–100% or near complete fusion.

### 2.1. Validity and Reliability of New Classification

A validation study of the novel midpalatal suture maturational stages classification was carried out by three experienced investigators (C.S., N.A., F.A.): an oral radiologist, an orthodontist, and an oral pathologist. Each had more than five years of experience interpreting CBCT scans for research and diagnostic purposes. Prior to the study, the investigators were shown the definitions and illustrations (Figure 1 and Figure 2) of the maturational stages via a PowerPoint presentation against a black background. Calibration took place using ten images, where all investigators independently classified the sutures, followed by a discussion of the different stages. For the validation phase, 30 images—randomly chosen to represent all maturational stages—were classified blindly by the three investigators in a controlled, dimly lit environment, using the same high-definition monitor. The same images were randomly reordered and reclassified by the investigators two weeks later to assess intra-rater reliability.

### 2.2. Statistical Analysis

The collected data were analyzed using SPSS version 25.0. Descriptive statistics were used for categorical and ordinal variables as frequencies and percentages. A normality check was not required, as the data being compared were categorical. The inter- and intra- examiner reliability and reproducibility was conducted to establish the validity and reliability of the new proposed MPS staging classification. Sensitivity and specificity tests were applied to detect MPS between the new proposed and standard classifications. Furthermore, the association between the possibility of an open MPS in the staging of MPS maturation using the new classification and gender across different age groups was examined using the chi-square test. Comparing the presence of an open MPS during MPS staging between males and females across four age groups, a binary logistic regression model was used to identify predictors of MPS maturation. A value of *p* < 0.05 was considered significant.

## 3. Results

The results derived from the study provides the following key information with regard to the maturation of MPS staging using the new classification and its predictability. A total of 168 CBCT images, spanning from 2018 to 2024, were investigated in this study.

The assessment of both intra-examiner (within the same reviewer) and inter-examiner (between different reviewers) reliability revealed outstanding agreement. This was quantified by weighted kappa coefficients ranging from 0.94 (with a 95% confidence interval [CI] of 0.89 to 0.99) down to 0.84 (95% CI, 0.78 to 0.89) (Table 1).

Table 2 presents the distribution of the study variables (age group, gender, current and proposed classification systems). The majority of CBCTs belonged to young adults aged 22–25 years (34.5%), followed by late adolescents aged 18–21 years (31.0%). Mid-adolescents (14–17 years) and early adolescents (10–13 years) accounted for 23.2% and 11.3%, respectively.

The study included 73 males (43.5%) and 95 females (56.5%), indicating a higher proportion of female participants. Based on the stages, the participants were classified into five stages (A–E), with the highest proportion in Stage C (33.9%) and the lowest in Stage A (6.5%). Within each stage, a further classification was made into patterns A and B. The most common group was Stage C—Pattern B (22.0%), followed by Stage D—Pattern B (14.9%) and Stage B—Pattern A (11.3%). The least common group was Stage A—Pattern A (3.0%).

**Figure 3:**
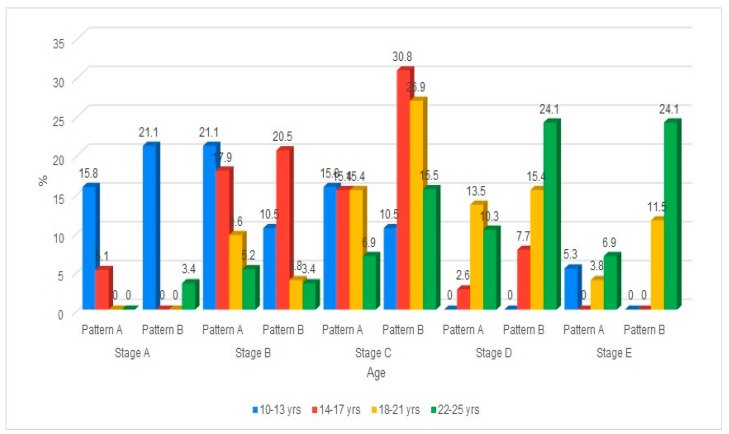
Cone Beam Computed Tomographic images of the New classification of Midpalatine Suture Staging with five stages and two patterns per stage.

The distribution of the maturation of the MPS by age group and gender is shown in Table 3. The maturation of the MPS progressed with age, with both males and females exhibiting a shift from early stages (A, B, C) in younger age groups to later stages (D, E) as age increased. In the maturation of the MPS, gender differences indicate that females tend to reach higher stages earlier than males, particularly evident in the 18–21 and 22–25 age groups. Early adolescence showed maturation of MPS stages of A, B, and C. Mid-to-late adolescents predominantly showed Stages B and C, with a gradual emergence of Stage D observed. However, in young adulthood, there was an increase in representation in Stages D and E (Figure 3).

The maturation of the midpalatal suture (MPS) was analyzed across males and females within four age groups (10–13, 14–17, 18–21, and 22–25 years). The analysis reveals a clear trend in which the possibility of maturation of the MPS decreases with increasing age across both genders. In the youngest age groups (10–13 and 14–17 years), a high proportion of both males and females were identified as having the possibility of maturation of the MPS, with percentages exceeding 88% and minimal differences between genders. In the 18–21 and 22–25-year groups, the proportion of individuals with the possibility of maturation of the MPS declined in both males and females, with the lowest rates observed in the oldest age group (22–25 years). Statistical comparisons within each age group demonstrated no significant differences between males and females regarding the possibility of maturation of the MPS, as indicated by *p*-values greater than 0.05. These findings suggest that while age is associated with a reduced likelihood of maturation of the MPS, gender does not significantly influence this association within any specific age group (*p* > 0.05).

The classification across five developmental stages (Stage A to Stage E) is further divided into two patterns (Pattern A and Pattern B), as proposed. The data was organized by age group (10–13, 14–17, 18–21, 22–25 years) and gender (male, female). In younger age groups (10–13, 14–17), more subjects are found in the earlier stages (A, B, C), with a larger spread across both patterns. As age increases (18–21, 22–25), a noticeable shift occurs, with more individuals progressing to later stages (D, E), particularly in Pattern B. The distribution between Pattern A and Pattern B within each stage shows some gender and age-related variation.

The maturation of the midpalatal suture (MPS) was analyzed across males and females within four age groups (10–13, 14–17, 18–21, and 22–25 years). The results showed that all males and 90.9% of females in the 10–13 age group displayed evidence of MPS maturation. Similarly, 88.2% of males and 95.5% of females aged 14–17 years showed a possibility of maturation of the MPS. Over half of the males (53.8%) and 84.6% of females in the age range of 18–21 revealed possibilities of maturation of the MPS. In contrast, less than half of the males (45.5%) and females (44.4%) showed the possibility of maturation of the MPS within the age range of 22–25 years. Hence, it is noted that individuals aged 10–17 exhibited high maturation rates in both males and females. The age range of 18–25 years showed a decline in maturation rates, especially among males. The gap between males and females is most pronounced in the 18–21 age group, although it is not statistically significant. However, maturation rates dropped to similarly low levels in both genders. All age groups showed a *p* > 0.05, indicating no statistically significant association between gender and maturation status in any age group.

A comparison of the proportion of individuals classified as having “Maturity” or “No maturity” using the current and proposed maturation of MPS classifications is detailed in Table 4. The current classification showed that 60.7% (102 out of 168) are classified as mature, while the proposed classification showed that 69.0% (116 out of 168) are classified as mature. The *p* < 0.001 indicates a statistically significant difference between the current and proposed classification systems in identifying “Maturity” versus “No maturity” of maturation of the MPS. This suggests that the proposed classification is more likely to classify individuals as mature compared to the current classification.

The predictors of maturation of the MPS are shown in Table 5. Both classifications indicate that age is a strong, statistically significant predictor of maturation of the MPS (*p* < 0.001). For the current classification, each unit increase in age increases the odds of maturation of the MPS by 3.57 times (95% CI: 2.28–5.58). For the proposed classification, a one-unit increase in age increases the odds of maturation of the MPS by 3.42 times (95% CI: 2.11–5.53), confirming its statistical significance. Hence, it can be noted that older age is consistently and strongly associated with a higher likelihood of maturity, regardless of classification type. Contrarily, gender was not found to be a significant predictor of maturation of the MPS in either the current classification (*p* = 0.419, Exp(B) = 0.74, 95% CI: 0.36–1.53) or the proposed classification (*p* = 0.114, Exp(B) = 0.55, 95% CI: 0.26–1.16). This suggests that gender has no significant effect on the maturation of the MPS.

A logistic regression analysis to predict a new classification based on age and gender indicates a good fitness of the new classification model. According to the observed and analyzed data, both classifications (standard reference and new) can rely on age (*p* < 0.001 and odds ratio 3.416) to predict the likelihood of maturation of the MPS compared to non-maturation, indicating that the new classification is a good fit. The new classification offers a higher possibility of detecting a midpalatine suture opening compared to the standard classification.

## 4. Discussion

The present study was conducted for applicability of the new classification to detect an open MPS and thus expandable sutures that can be managed without surgery. The highest prevalence of the samples (60.6%) was in the potentially expandable, non-surgical expansion categories (Stage A: 11, B: 34, C: 57). The potentially non-expandable stage that would require surgical intervention accounted for up to 39.3% of the study samples (Stage D and E). Stage C, with 33.9%, was the single most common stage in both classifications that were partially open/partially fused and possibly had high resistance to expansion, with a definite possibility of expansion. The new classification subcategorizes each stage into Pattern A and Pattern B for greater diagnostic accuracy in detecting stages with patterns that utilize non-surgical expansion. The possibility of detection of an open MPS was highest in the younger age groups (100%) (10–13-year old males) and 90% in 14–17-year old females. The likelihood of open MPS sharply decreased with increasing age, as shown in the results, with 33% in females and 36.4% in males in the 22–25-year-old group. The above results reinforced the notion that age is a determining factor in MPS maturation. Gender was not a determining factor in MPS maturation, as the Chi-squared test revealed no significance between gender and the likelihood of detecting an open MPS. The few deviations from the above are likely due to chance. The standard classification and the new classification thus relied on age as a predictable variable, with the new classification displaying increased sensitivity in detecting open MPS.

The midpalatine suture region is one of the key areas where the biological process of suture maturation can be harnessed for the prevention and treatment of malocclusion due to underdevelopment of the maxilla, resulting in size/shape discrepancies in the maxillary arch (transverse maxillary deficiency). Maxillary transverse deficiency (MTD) can cause various dentofacial abnormalities, including tooth crowding, anterior tooth protrusion, posterior tooth crossbite, mouth breathing, and obstructive sleep apnea. It is also associated with various abnormalities such as cleft lip/palate and craniosynostosis [8]. MTD cannot be resolved over time during the individual’s development. Therefore, an early diagnosis and intervention are imperative. Treatment of such conditions involves maxillary expansion. One of the key factors in the treatment plan is the stage of maturation of MPS and the patient’s age.

The present study evaluated the maturation of the midpalatine suture by exploring new subcategories of classification and comparing them with the staging of the previous MPS classification by. In the present study, the majority of the sample consisted of young adults or late adolescents, which can be attributed to the most common age group seeking dental care within the specified age range selected for this study. Additionally, the female gender was more prevalent than the male gender in the study population, possibly due to the greater proportion of females seeking dental care, which may be attributed to higher oral health literacy and oral care [9].

The midpalatal sutural fusion was thought to be complete by the age of 15 to 19 years. Unsurprisingly, the possibility of RME was previously considered only until 16 years of age, as with older age, the midpalatal suture and adjacent articulations start to fuse and become more rigid, leading to a higher resistance to expansion forces [10]. During the period of late adolescence and adulthood, extra force is indispensable to open the MPS due to its increased degree of interdigitation. Consequently, after the age of 16, MARPE or SARPE was frequently used to overcome these limitations by surgically releasing the interdigitated suture prior to maxillary expansion [11]. Some studies suggest that RME should be performed before puberty, based on the above observation [12,13]. However, contrasting results of fusion of the MPS were found in studies across various age groups, with variations observed up to the age of 71 years, indicating that age may not be a completely reliable factor for MPS maturation [10,14,15]. These findings were noted in the present study, where there were cases in which age and the maturation of the MPS were inconsistent. In this study, it was also noted that although younger individuals are more likely to represent immature stages of the MPS, a notable share of young adults and late adolescents may be in stages that are amenable to non-surgical expansion. Although the majority of the scan age groups could predict the MPS’s maturation and staging, it was interesting to find that in some cases, the MPS staging differed from that predicted by age. The stages of fusion of the midpalatal suture were found to vary significantly with age. These findings were similar to those of Angelieri et al. (2013) and Haghanifar et al. (2017) [2,4]. However, further analysis showed that age was a significant predictor of MPS maturation in this study, while gender did not play a significant role in maturation of the MPS. Gender may not be a significant factor, as the closure of palatine sutures is influenced by various factors, including age, vascularity of the region, the genetic composition of the individual, hormonal and locoregional conditions, and other factors [16].

CBCT is the most widely used imaging technology in dentistry for assessing the maturation of the MPS. In the previous classification of maturation of the MPS using CBCT, Stage C was found to be the most prevalent [2]. This finding is noteworthy, as at this stage, maxillary expansion is still achievable without surgical intervention. Although this classification can explain the maturation stages, variations were observed that did not fit into specific stages of maturation [4,7]. Additionally, the results from a cohort study evaluating 63 adolescent patients in the pre- and post-phases of adolescence, using pre- and post-expansion with RME, assessed the applicability of the classification. The study concluded that this classification requires further research and validation and did not support the efficacy of this classification in predicting changes in maturation of the MPS [17].

The proposed classification provided more details for maturation staging, with the most prevalent being Stage C—Pattern B (22.0%), followed by Stage D—Pattern B (14.9%) and Stage B—Pattern A (11.3%). The distinguishing ability of these patterns within the same stage enables diagnostic precision. Subtle variations in maturation within the same stage, represented as Pattern A and Pattern B, can influence the extent of resistance to maxillary expansion in the MPS. The prognosis of RME is determined by the maturation of the MPS and the bone strength at this level, along with the multitude of sutures in the facial skeleton [18]. The choice of RME, such as conventional RME, MARPE, or SARPE, and the differences in design of these RME appliances, their confirmation, and the techniques directed to achieve specific treatment outcomes rely on the maturation of the MPS [19]. Hence, for RME, maturation of the MPS should be evaluated as part of the treatment plan rather than relying solely on age.

The new classification of MPS maturation using CBCT also demonstrated a higher detection rate of MPS maturity compared to the previous classification. The higher detection rate of MPS maturation in identifying an open MPS indicates that the new system of classification showed improved sensitivity in identifying maturity stages and thus has greater diagnostic accuracy. The applied aspect of the new classification emphasizes that individual or personalized suture assessment is crucial in treatment planning for maxillary expansion, rather than relying on standard chronological age-based treatment planning.

Thus, in stages and patterns of MPS maturation according to the new classification, where the MPS is open or partially ossified from Stage A to Stage D, Pattern A, non-surgical maxillary expansion techniques can be considered including RME, SME, and MARPE [3,4,20,21,22]. In the previous classification, this possibility was limited to Stage C [2]. Additionally, Stage D—Pattern B and Stage E—Pattern A in the new classification indicated the possibility of maxillary expansion results being feasible, confined to the maxillary segment alone of the hard palate. For Stage E—Pattern B, where ossification is complete, surgical techniques such as SARPE or Maxillary Le Fort I osteotomy can be considered [23,24].

The most compelling evidence that the new classification is superior to the previous classification in terms of clinical applicability is the extension of stages where non-surgical maxillary expansion is considered achievable, and the identification of a larger sample of mature patients who can benefit from the aforementioned less invasive maxillary expansion techniques. The new classification also guides maxillary expansion, even in stages such as D and E, where limited maxillary expansion is still attainable, confined to the maxillary segment of the hard palate. The new classification allows clinicians to achieve non-surgical, targeted results in maxillary expansion. The new classification thus offers more detailed predictions an open MPS compared to the previous classification, which treats cases deemed mature, making it a superior clinical tool compared to the previous method of assessing MPS maturation.

Limitations of the study included that the research was conducted as a single center study; interobserver bias, the investigators required calibration twice to achieve good inter-examiner agreement and reliability, as well as to standardize the detection of staging patterns and absence of clinical validation. The study did not compare the MPS maturation with the facial growth patterns of the face.

Prospective studies are necessary to determine whether the new patterns identified in the staging of the MPS in the new classification translate into measurable outcomes in treatment planning, treatment outcomes, and stability, thereby evaluating the efficacy and validity of the proposed new classification. A larger sample size will definitely increase the validity and applicability of the new classification. Further, studies integrating artificial intelligence training models to detect the maturation stages of the MPS will ease the detection of MPS and be beneficial. Recently, an artificial intelligence model has been tried for maturation of the MPS using 2D convolutional models, as proposed by the previous classification) [2,25]. The study also states that if future studies challenge the classification, the validity of the aforementioned study will be affected. Thus, the present study and the new classification should be explored as a new scaffold for AI training and the detection of open sutures.

## 5. Conclusions

The study observed a clear progression of maturation, as indicated by midpalatine suture staging, and this finding was valid in both classifications. Age was a strong predictor of the maturation staging in both classifications. In the new classification, each stage was divided into Pattern A and Pattern B, with the most common patterns being Stage C—Pattern B and Stage D—Pattern A, which provides a crucial diagnostic differentiation, suggesting a new classification of superior choice. The new classification demonstrated increased sensitivity in detecting midpalatine suture maturity. This improved accuracy enables more reliable staging and assessment, thereby enhancing the potential to guide palatal expansion decisions where previous classifications have shown limitations. The present study highlights the role of CBCT-based suture staging in personalized interventions, establishing a new classification system that improves clinical decision-making. The results are preliminary and require multicenter and prospective validation.

## Figures and Tables

**Table 1:** Sensitivity and specificity midpalatine suture staging of the new classification versus previous classification.

Sensitivity and Specificity MPS Stages					
			Previous Classification		Total
			Maturity	No Maturity	
New classification	Maturity	Count	102	14	116
		% within previous classification	100.0%	21.2%	69.0%
	No maturity	Count	0	52	52
		% within previous classification	0.0%	78.8%	31.0%
Total		Count	102	66	168
		% within previous classification	100.0%	100.0%	100.0%

**Table 2:** Distribution of study variables, their frequency, and percentage.

Study Variables (*n* = 168)		*n*	%
Age	10–13 (Early adolescence)	19	11.3%
	14–17 (Mid-adolescence)	39	23.2%
	18–21 (Late adolescence)	52	31.0%
	22–25 (Young adults)	58	34.5%
Gender	Male	73	43.5%
	Female	95	56.5%
Current classification of maturation of MPS	Stage A	11	6.5%
	Stage B	34	20.2%
	Stage C	57	33.9%
	Stage D	39	23.2%
	Stage E	27	16.1%
Proposed classification of maturation of MPS	Stage A—Pattern A	5	3.0%
	Stage A—Pattern B	6	3.6%
	Stage B—pattern A	19	11.3%
	Stage B—pattern B	14	8.3%
	Stage C—pattern A	21	12.5%
	Stage C—pattern B	37	22.0%
	Stage D—pattern A	14	8.3%
	Stage D—pattern B	25	14.9%
	Stage E—pattern A	7	4.2%
	Stage E—pattern B	20	11.9%

**Table 3:** Maturation of midpalatine suture staging based on previous classification (2013).

Maturation of Midpalatine Suture Staging												
Age	Gender	Stage A		Stage B		Stage C		Stage D		Stage E		Total
		*n*	%	*n*	%	*n*	%	*n*	%	*n*	%	
10–13	Male	4	50.0	3	37.5	1	12.5	0	0.0	0	0.0	8
	Female	3	27.3	4	36.4	3	27.3	0	0.0	1	9.1	11
14–17	Male	0	0.0	7	41.2	8	47.1	2	11.8	0	0.0	17
	Female	2	9.1	8	36.4	10	45.5	2	9.1	0	0.0	22
18–21	Male	0	0.0	4	15.4	8	30.8	7	26.9	7	26.9	26
	Female	0	0.0	3	11.5	14	53.8	8	30.8	1	3.8	26
22–25	Male	2	9.1	2	9.1	4	18.2	7	31.8	7	31.8	22
	Female	0	0.0	3	8.3	9	25.0	13	36.1	11	30.6	36

**Table 4:** Comparison of maturation of Midpalatine Suture (MPS) between current and proposed classification.

Maturation of MPS—Current and Proposed Classification				
Current Classification		Proposed Classification		*p*
Maturity	No maturity	Maturity	No maturity	<0.001
102 (60.7%)	66 (39.3%)	116 (69%)	52 (31%)	

**Table 5:** Predictors of maturation of Midpalatine Suture (MPS).

Predictors of Maturation of MPS									
		B	S.E.	Wald	df	Sig.	Exp(B)	95% CI for Exp(B)	
								Lower	Upper
Previous classification	Age	1.272	0.228	31.221	1	0.000	3.569	2.284	5.577
	Gender	−0.297	0.367	0.654	1	0.419	0.743	0.362	1.526
	Constant	−3.849	0.890	18.720	1	0.000	0.021		
Proposed classification	Age	1.228	0.246	25.001	1	0.000	3.416	2.110	5.529
	Gender	−0.601	0.380	2.501	1	0.114	0.548	0.260	1.155
	Constant	−3.705	0.937	15.645	1	0.000	0.025		

Variable(s) entered on step 1: Age, Gender.

## Data Availability

The data presented in this study are available on request from the corresponding author due to (regional data sharing regulation).
